# Bird Species Detection Net: Bird Species Detection Based on the Extraction of Local Details and Global Information Using a Dual-Feature Mixer

**DOI:** 10.3390/s25010291

**Published:** 2025-01-06

**Authors:** Chaoyang Li, Zhipeng He, Kai Lu, Chaoyang Fang

**Affiliations:** 1 Jiangxi Protected Area Construction Center, Nanchang 330006, China; 2 Key Laboratory of Poyang Lake Wetland and Watershed Research, Ministry of Education, Jiangxi Normal University, Nanchang 330022, China; 3 School of Geography and Environment, Jiangxi Normal University, Nanchang 330022, China

**Keywords:** bird detection, feature extraction, class imbalance, deep learning

## Abstract

Bird species detection is critical for applications such as the analysis of bird population dynamics and species diversity. However, this task remains challenging due to local structural similarities and class imbalances among bird species. Currently, most deep learning algorithms focus on designing local feature extraction modules while ignoring the importance of global information. However, this global information is essential for accurate bird species detection. To address this limitation, we propose BSD-Net, a bird species detection network. BSD-Net efficiently learns local and global information in pixels to accurately detect bird species. BSD-Net consists of two main components: a dual-branch feature mixer (DBFM) and a prediction balancing module (PBM). The dual-branch feature mixer extracts features from dichotomous feature segments using global attention and deep convolution, expanding the network’s receptive field and achieving a strong inductive bias, allowing the network to distinguish between similar local details. The prediction balance module balances the difference in feature space based on the pixel values of each category, thereby resolving category imbalances and improving the network’s detection accuracy. The experimental results using two public benchmarks and a self-constructed Poyang Lake Bird dataset demonstrate that BSD-Net outperforms existing methods, achieving 45.71% and 80.00% mAP50 with the CUB-200-2011 and Poyang Lake Bird datasets, respectively, and 66.03% AP with FBD-SV-2024, allowing for more accurate location and species information for bird detection tasks in video surveillance.

## 1. Introduction

Bird species detection is the process of identifying the location and species of birds in their natural environment, which is critical for many real-world applications. It can be used in environmental monitoring to understand changes in bird populations [1], as well as to assess biodiversity through bird species counts [2]. In addition, accurate bird species identification is critical for the preservation of endangered species and biodiversity [3]. Unfortunately, the inherent characteristics of bird size and inter-species similarity, as well as class imbalance, present challenges to algorithm design.

Currently, deep learning technology has achieved remarkable success using object detection algorithms, such as the faster region-based convolutional neural network (Faster R-CNN) [4], you only look once (YOLO) model [5], and generalized focal loss (GFL) model [6], to detect animal objects. The existing bird species detection methods can be divided into two types: acoustic feature-based and visual feature-based. The acoustic feature-based method converts bird sounds into spectrograms and then uses neural networks to extract features from the spectrograms and detect bird species [7,8,9]. Xie et al. [10] proposed a multi-view fusion feature method for bird voice recognition that combines features extracted by convolutional neural networks, called “depth features”, and manually engineered features; the results indicated that this method could achieve a 95% accuracy for 16 bird species. Kahl et al. [11] developed BirdNet, a deep neural network capable of identifying 984 bird species in North America and Europe based on sound. BirdNet is publicly available. However, the natural environment contains a variety of noises, including animal sounds and wind noise, which reduces the accuracy of bird species detection. Unlike acoustic feature-based methods, visual feature-based methods allow birds to be observed directly from images, with local, global, and color features learned from the images to detect bird species. BirdGuides [12], a pioneer in this field, created an image-based bird detection system that can identify 500 of North America’s most common bird species. Huang et al. [3] developed a convolutional neural network incorporating skipped connections, with direct pathways between earlier and later layers, to improve gradient flow and facilitate learning. They annotated 27 species of birds collected on the Internet, and the convolutional neural network model achieved an accuracy of 95.37% in classifying 27 species of birds. However, the method only stacks convolutional layers and cannot accurately identify birds of similar species. Ferreira et al. [13] developed a method for identifying individual birds using data on captive birds. They selected an iteratively pre-trained convolutional neural network to classify three species of birds, achieving a recognition accuracy of 90%. However, this method mostly focuses on individual identification and can face certain challenges when applied to bird species identification in the natural environment. Lin et al. [14] introduced a fusion module structure that transferred global feature information to the local feature extraction module and used skipped connections to facilitate the interaction between global and local information. To verify the performance of the model, they acquired and collated bird image data from the Internet to construct a Fujian bird dataset, and the experimental results showed that the accuracy of bird recognition was 94.8%. Yi et al. [15] introduced the Res2Net-CBAM module, which is based on YOLOv5, to improve the receptive field and channel characteristics of each network layer while increasing the model’s sensitivity to important information. The classification results using the CUB-200-2011 benchmark dataset demonstrated that the improved model achieved a 1.2% improvement over the baseline, having an accuracy of 86.6%. Nonetheless, it should be highlighted that this study only focused on image classification without addressing the detection of birds’ locations. Liu et al. [16] proposed a cross-modal semantic alignment and feature fusion (CSAFF) network that incorporates motion behavior information obtained from bird trajectories, such as motion direction and velocity, as an additional cue to improve recognition accuracy. This information is represented as temporal motion images, which are used as one-dimensional sequences to provide discriminative features. To verify the effectiveness of the proposed method, they collected biomimetic drone and bird image data in the natural environment and evaluated the method’s performance. The results showed that the recognition accuracy of the bionic drone reached 95.25%. However, this method was not evaluated on a bird benchmark dataset. Liang et al. [17] employed self-supervised predictive convolutional attention blocks to enhance key feature extraction in a YOLOv5-based framework. Their approach improved the network’s expressiveness by employing a multi-branch architecture that integrates features from different receptive fields. Kumar et al. [18] used a transfer learning method combined with a hybrid hyperparameter optimization scheme to achieve the optimal hyperparameter selection based on the weights of pre-trained deep learning models. The experimental results using the CUB-200-2011 bird benchmark dataset showed that the fine-tuned EfficientNetB0 model had an accuracy of 99.12% for the classification of bird species. Still, it should be noted that they only classified bird images but did not address the problem of bird location detection. These methods produce excellent results, demonstrating the importance of efficient feature extraction and contextual information aggregation in bird species detection.

However, there are still some challenges with the existing methods. First, most algorithms do not consider the similarity of bird species’ local structures [15,17,19], such as the resemblance between the beaks of different birds. Nevertheless, it is not uncommon for different bird species to share similar local structures. Therefore, there are similarities in the local features of network learning, which leads to misclassification of classes. It is worth noting that global context information facilitates the identification of locally similar features. This observation prompted us to include attention mechanisms in the network structure. Currently, the transformer mechanism [20,21,22] effectively expresses global context information. Carion et al. [21] proposed the detection transformer (DETR), which uses CNNs to learn local information and an encoder–decoder transformer to capture global features. Particularly, the DETR infers the global contextual relationships in an image and directly predicts results in parallel. The results indicated that the DETR achieved the AP50 value of 62.4% with the COCO benchmark dataset. However, training the baseline model for 300 epochs on 16 V100 GPUs took three days. Hatamizadeh et al. [23] introduced the global context vision transformer (GC ViT), which combines a global context self-attention module with standard local self-attention to model both long- and short-range spatial interactions effectively. The results showed that the GC ViT achieved a 58.3% APbox with the COCO benchmark dataset. Zhao et al. [24] proposed the real-time detection transformer (RT-DETR), which constructs an efficient hybrid encoder through attention-based intra-scale feature interaction and CNN-based cross-scale feature fusion. The results demonstrated that the RT-DETR (ResNet50) achieved the mAP50 value of 53.1% with the COCO benchmark dataset. However, the memory and computational costs associated with directly using the transformer mechanism have increased exponentially.

Second, bird species detection often encounters class imbalances [25]. This is due to the survival of the fittest principle in nature, as well as to the influence of human factors, which causes an imbalance in the data, resulting in inconsistent frequency and size of each bird during the training phase [26]. This presents a challenge for bird species detection. A common solution to this problem is to downsample the head category while oversampling the tail category [27,28] or to balance sample importance (using category or image weights) [29,30,31]. However, even the current state-of-the-art methods face performance degradation in the tail category [18,32,33].

Third, there are insufficient high-quality datasets for bird species detection. In the object detection task, the COCO [34] image recognition dataset encourages the advancement of recognition technology. Although COCO officially provides an application programming interface (API) for filtering custom categories for training, it only covers a limited number of bird images and does not include species classification. In addition, the existing methods generate datasets by collecting bird image annotations from the network. However, there are certain differences between these bird images and video surveillance images, which makes the algorithm unable to meet the practical application needs.

In this paper, we focus on solving the above problems. First, to address the issue of local structural similarity in birds, we propose a dual-branch feature mixer capable of dynamically learning local and global context information. Specifically, it employs global attention and deep convolution to extract features from dichotomous feature segments, thereby expanding the network’s receptive field and achieving a strong inductive bias, allowing the network to distinguish between similar local details. Unlike the previous approach, we sparsely combine local details and global information. Second, to address the problem of class imbalance in the training process, we propose a prediction balance module to assist the model in producing more robust prediction results. This balance depends on the number of pixels and the variance variation for each category. This is demonstrated by assigning more changes to the tail class and fewer changes to the head class. With this balance, we close the gap between feature regions from different categories, which results in a more balanced representation. Finally, a bird dataset is constructed for Poyang Lake that can be used for species detection tasks. Our BSD-Net performed well in detecting bird species using two public bird benchmark datasets as well as the self-constructed Poyang Lake Bird dataset.

## 2. Methods

In this section, we begin with a problem statement. We then discuss the proposed BSD-Net and its two main modules: the dual-branch feature mixer (DBFM) and the prediction balance module (PBM).

Problem Statement: Given an image, the objective is to identify the location and species of each bird. We train a deep learning model, also known as BSD-Net, with parameters by minimizing the difference between the predicted bounding box position and bird species and the ground truth.

### 2.1. Network Architecture

**Figure 1:**
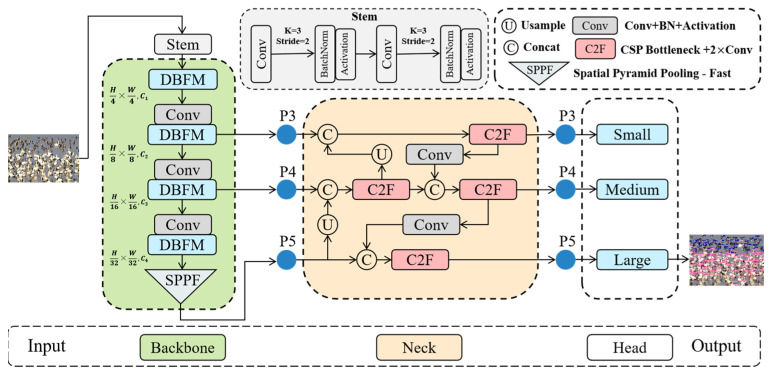
The overview of the proposed BSD-Net. First, the backbone, which includes a dual-branch feature mixer, is used to extract features from the input image. Then, in the neck module, multi-scale feature fusion is performed to enhance the feature representation capabilities. Finally, in the head module, multi-scale features are used to generate detection box position and classification results.

Figure 1 depicts our BSD-Net’s network architecture, which is based on YOLOv8 [35]. In the backbone network, the stem consists of two convolutional layers, batch normalization, and a SiLU activation function that downsamples the input image to produce a feature map with dimension $\left(\frac{H}{4}\times \frac{W}{4}\right)$ and channel count C1. The dual-branch feature mixer and convolution are then stacked between layers to extract local and global features, followed by feature fusion using the SPPF module. In the neck section, the PAFPN method is used to tandem and upsample the P3, P4, and P5 layers, allowing for feature propagation across scales through convolution and C2F. The head section generates detection and classification results at three different scales: large, medium, and small. The following sections will provide more information about the BSD-Net structure.

### 2.2. Dual-Branch Feature Mixer

**Figure 2:**
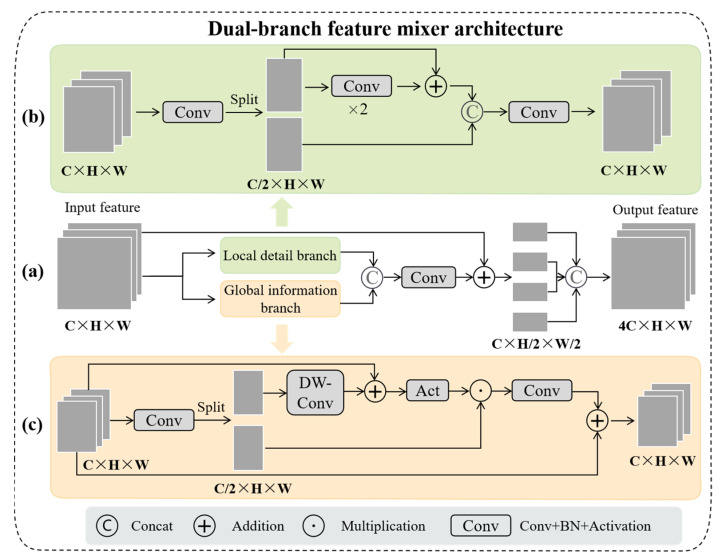
The overview of the dual-branch feature mixer. (**a**) A feature map $X\in R^{C\times H\times W}$ is first input into the local detail branch and global information branch. The output features are concatenated along the feature dimension and undergo a 1 × 1 convolution for channel information mixing. Then, the mixed features are added to the input features in the feature channels, split into four subregions, and, finally, mixed again in the feature channels; (**b**) the local detail branch employs the C2F module from the YOLOv8 model to extract local features; (**c**) the global information branch evenly divides the input features into two sub-feature maps along the channel dimension. The depthwise separable convolution (DW-Conv) and skip connections are used to preserve the spatial resolution information of the image. The branches are merged through element-wise multiplication, and a 1 × 1 convolution is applied to refine the global information.

Many previous methods used hybrid models that combined convolutional neural networks (CNNs) and transformers to improve the network’s ability to represent global contextual information [36,37]. However, in the face of large-scale image data, transformer requires massive computing resources and memory to comprehend global contextual information. Recent studies have demonstrated that gated multilayer perceptron (gMLP) can improve models’ expression ability in natural language processing tasks, and we discovered that gated MLP can also interact with the spatial context of global context information in visual tasks [38]. Based on this observation, we propose a dual-branch feature mixer (DBFM) that dynamically learns local and global contextual information while consuming minimal computational resources, allowing the network to distinguish between similar local details. Figure 2 depicts the overall workflow for the proposed dual-branch feature mixer.

Specifically, the C2F module in the YOLOv8 is used in the local details branch because of its powerful local detail extraction capabilities. For the global information branch, two branches $F_{1}$ and $F_{2}$ are created after passing through the convolutional layer from the input feature map F. The $F_{1}$ branch uses the depth separable convolution to extract the spatial information of the image and performs gradient update in the way of residual addition during the training process. As a result, the computational resources are reduced, and the network performance is improved. Then, the information of each level is controlled by the GELU activation function, which is multiplied by the $F_{2}$ branch to capture the global context information through 1 × 1 convolution. Finally, the input feature map is output in the form of residual addition. The mathematical expression for global information extraction is as follows:

(1) $$F_{1},F_{2}=Split(SiLU(BN2d({Conv}_{1\times 1}(F))))$$

(2) $$GI={Conv}_{1\times 1}({(GELU(DWConv}_{3\times 3}(F_{1}+F)))\times F_{2})+F$$

where F represents the input feature map of the previous layer, $F_{1}$ and $F_{2}$ are, respectively, F divided by the feature channels 1 and 2, and GI denotes the extracted global context information. Compared to MLP, the gating mechanism in global information extraction can capture the correlation of adjacent features and express the global features and interrelationships on each pixel, thereby increasing the network’s expressiveness.

Finally, we concatenate the local details LD and the global context information GI in the feature channel and mix the channel information using 1 × 1 convolution, which is expressed as follows:

(3) $$F^{d}=SiLU(BN2d({Conv}_{1\times 1}(Concat(LD+GI))))$$

where LD represents a local detail feature, GI is global context information, and $F^{d}$ denotes a dual-branched feature.

Furthermore, the conventional downsampling method typically employs a 3×3 convolution with a step length of 2, which has a large receptive field and can ensure sampling efficiency; however, for avian features of small targets, a small convolutional kernel can extract the refined features. Therefore, the dual-branched feature $F^{d}$ is divided into four sub-regions $\left(F_{0}^{d},F_{1}^{d},F_{2}^{d},F_{3}^{d}\right)$, each of which is of size *H*/2, *W*/2, *C*, and then mixed in the feature channels, which is mathematically expressed as follows:

(4) $$F^{dbfm}=Concat(F_{0}^{d},F_{1}^{d},F_{2}^{d},F_{3}^{d})$$

where $F^{dbfm}$ is the feature extracted by the dual-branch mixer.

### 2.3. Prediction Balance Module

**Figure 3:**
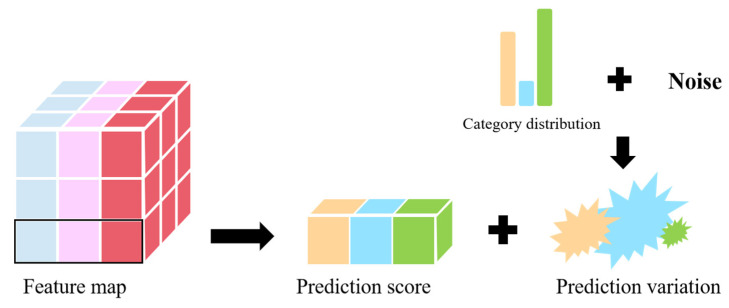
The block diagram of the PBM. The prediction score represents the predicted scores for each class output by the model’s head module, where different colors indicate different classes (e.g., three classes). Prediction variation is generated based on class distribution and noise perturbation, where the scale of noise perturbation is inversely proportional to the class distribution. This approach provides a more balanced representation of the feature space across classes.

The dual-branch feature mixer allows the network to fully learn the image’s fine-grained features. However, because the size and number of birds in the natural scene vary, the number of samples in the training data category is imbalanced. The network may overfit the features of the head class while compressing the features of the tail class in a small area, leading to overconfidence in the head class’s prediction results. Inspired by [39], we propose a new prediction equilibrium module for balancing the differences between different category feature regions. Figure 3 provides a more intuitive understanding of the prediction balance module.

Specifically, during the training process, we obtain the pixel values of each category based on the size of each image and the bounding box information and then calculate the class frequency vector *Pix* using Equation (5). Each category in the class frequency has an inverse relationship with the pixel value. One intuitive way to accomplish this is to balance the class prediction score based on class frequency. However, bird object detection is an instance recognition task, and a simple balancing strategy can result in overfitting issues. As a result, we suggest increasing the variation in the fixed class frequency, as expressed in Equation (6):

(5) $${Pix}_{k}=\log\frac{\sum_{i=0}^{C-1}p_{i}}{p_{k}}$$

(6) $${Variation}_{k}=\frac{{Pix}_{k}}{{max}_{i=0}^{C-1}{Pix}_{i}}\left|\delta \left(\sigma \right)\right|$$

where $p_{k}$ represents the pixel value of the class k, ${Pix}_{k}$ is the inverse change of the class frequency, $\delta \left(\sigma \right)$ denotes the Gaussian distribution with a mean of 0 and a variance of σ, and ${Variation}_{k}$ is the change according to the class frequency. Finally, we add variation to the prediction score to help balance the gap between the different categorical feature regions, which is expressed as follows:

(7) $${Pred\_scores}_{k}={Pred\_scores}_{k}+{Variation}_{k}$$

where ${Pred\_scores}_{k}$ is the predicted score for each category, and ${Variation}_{k}$ is the predicted change for each category. With this balance, we aim to balance the feature representation space of different classes. Notably, this balance persists in the training phase and is discarded in the inference phase to help the network achieve robust predictions.

## 3. Poyang Lake Bird Dataset

Currently, there are some datasets with birds as objects, such as the Drone2021 [40] and MVA2023 Birds dataset [41]. However, due to the unique nature of the task, they are primarily used in UAV collision avoidance. The published benchmark datasets for bird species detection are CUB200-2011 [42] and FBD-SV-2024 [43]. The CUB200-2011 dataset was the first bird species detection dataset, with 11,788 images of 200 bird species. However, the high definition of each image in this dataset, as well as the fact that a single bird occupies the entire image, are inconsistent with actual video surveillance images. The FBD-SV-2024 dataset consists of 483 video clips totaling 28,694 frames, of which 23,833 contain 28,366 bird instances. However, the maximum number of pixels occupied by the bird targets in this dataset is approximately 96 × 96 pixels.

**Figure 4:**
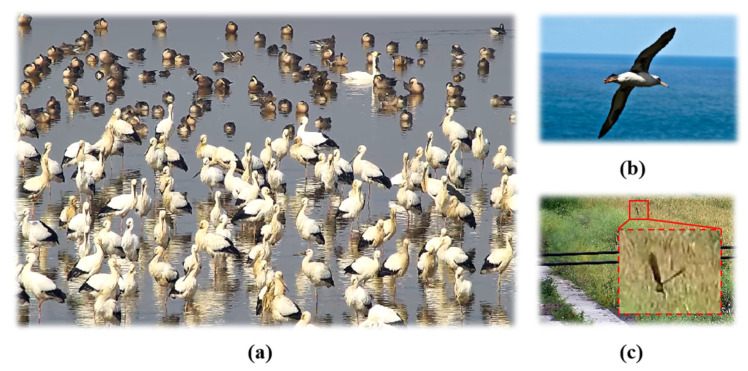
(**a**) Bird dataset of the Poyang Lake; (**b**) CUB-200-2011 dataset; (**c**) FBD-SV-2024 bird dataset.

Every year, many migratory birds visit Poyang Lake, but there is a lack of relevant bird species datasets for bird species detection, and neural networks trained with the benchmark datasets perform poorly when applied directly to video surveillance. Therefore, it is critical to construct a bird species identification dataset that is compatible with video surveillance and includes multiple birds in a single image. To solve this problem, we constructed the Poyang Lake Bird dataset, which is consistent with video surveillance observations, and the neural network trained on this dataset can be directly generalized to the real-world environment. Figure 4 depicts an example of the Poyang Lake Bird dataset (CUB-200-2011 and FBD-SV-2024).

To ensure the dataset’s quality, we used automated video surveillance footage in conjunction with manual screening to collect bird data from Poyang Lake. Next, we used the *Chinese Bird Field Handbook* as a reference for bird species labeling and then used the third-party software “Label Studio1.7” to label the bounding boxes and species in all images, saving the labeling format as COCO. It is important to note that for birds of uncertain species, we collaborated with ornithology experts to ensure the accuracy of the annotated images. In addition, we excluded birds from ten images to reduce the effect of category imbalance on the neural network generalization ability. Finally, we created a dataset of birds in Poyang Lake, including 34 species of birds that have appeared in the lake in recent years, totaling 12,545 images, and divided it into a training set and a test set in a 7:3 ratio for each bird.

## 4. Experiments and Analysis

Our proposed BSD-Net was evaluated on the publicly available benchmark datasets CUB-200-2011 and FBD-SV-2024, as well as on the self-constructed Poyang Lake Bird dataset. In addition, the two proposed modules and hyperparameters were examined to verify the effectiveness of the designed modules.

### 4.1. Description of the Datasets

CUB-200-2011 dataset: The CUB-200-2011 dataset [42] is a fine-grained benchmark dataset of birds published by the California Institute of Technology. It contains 11,788 images of 200 bird species, with each image providing image class tag information, such as bird bounding box, key site, and attribute information. Among them, 5994 images were used as the training set, and 5794 images were used as the validation set. The dataset is high-quality and diverse. It is important to note that this dataset contains large targets, with only one bird species per image, and was appropriate for assessing the model’s performance in identifying large targets.

FBD-SV-2024 dataset: The FBD-SV-2024 dataset [43] extracts bird data from video frames and uses the open-source tool labelImg to label the categories and bounding boxes of bird objects in images. The annotation process was divided into three rounds, each handled by a different person and cross-checked to ensure the annotation’s quality. The training set consisted of 23,979 images, while the validation set included 4715 images. It is worth noting that the birds in this dataset occupy a very small number of pixels, which was appropriate for assessing the model’s accuracy in identifying small targets.

Poyang Lake Bird dataset: This dataset includes 12,545 images of 34 bird species, each with bounding box information and species categories. The training set consisted of 8768 images, while the test set comprised 3777 images. The dataset is consistent with the video surveillance images, and each image contains multiple targets, making it appropriate for evaluating the model’s detection and generalization ability in multi-target scenarios.

### 4.2. Implementation Details and Metrics

In our experiments with two datasets, CUB-200-2011 and Poyang Lake Bird dataset, we used a single NVIDIA GeForce RTX 4090 Laptop (16 GB) GPU based on the PyTorch framework. We did not use pre-training weights to ensure a fair comparison and fully reflect the network architecture’s feature learning ability. Instead, we trained 100 epochs from scratch, set the batch size of training and validation to 16, set the initial learning rate to 0.01 and the momentum to 0.937, fixed the input image size to 640 × 640, and trained and updated the model weights using the SGD optimization algorithm. With the FBD-SV-2024 dataset, we used the FBOD-BMI experimental setup and an image size of 672 × 384, with the remaining settings consistent with those used for the other two datasets. During training, we performed image data augmentation using random zoom, panning, and mosaic, with mosaic data augmentation disabled for the last ten epochs.

To evaluate the detection results, Recall, Precision, AP, and mAP were used as evaluation metrics. The mathematical equations for Recall, Precision, AP, and mAP are as follows:

(8) $$Recall=\frac{TP}{TP+FN}$$

(9) $$Precision=\frac{TP}{TP+FP}$$

(10) $$AP=\sum_{n}(R_{n}-R_{n-1})P_{n}$$

(11) $$mAP=\frac{1}{C}\sum_{c=1}^{c}{AP}_{c}$$

where TP represents a true positive, FP denotes a false positive, FN is a false negative, Recall represents the recall, Precision denotes precision, $R_{n}$ and $P_{n}$ are the recall and precision of point n, respectively, AP represents the average accuracy of each category, C is the number of categories, and mAP denotes the average accuracy of all categories and is used to measure the detection performance of the network.

### 4.3. Experimental Results and Analysis

Evaluation on the CUB-200-2011: Table 1 presents the quantitative results for BSD-Net and other advanced networks using the CUB-200-2011 dataset. The experimental results demonstrated that BSD-Net outperformed other networks. Notably, BSD-Net had a 4.51% higher mAP50 than Yolov8m. Yolov8m had a C2F module for efficient object detection, but it lacked the ability to aggregate local and global context information. BSD-Net, on the other hand, proposes a new dual-branch feature mixer that can effectively aggregate, pixel-by-pixel, local and global context information from the input image, while also discriminating local similar features.

Based on the quantitative results obtained using the transformer method (RT-DETR), the detection effect was not ideal, which could be attributed to the absence of pre-training weights and hyperparameter settings. The pre-trained weights provided good initial weights to the model and learned common features from large-scale datasets, which accelerated the fitting process of the model to new datasets. In addition, hyperparameters, such as learning rate, batch size, and optimizer, could significantly affect the training effect and final performance of the model. For a fair comparison, this study performed training using the hyperparameters provided by the method proposer.

Evaluation on the FBD-SV-2024 dataset: Table 2 presents the quantitative performance of BSD-Net and other advanced networks using the FBD-SV-2024 avian dataset. The experimental results showed that BSD-Net outperformed other networks. It is worth noting that BSD-Net’s AP was 4.95% higher than YOLOv8l’s. This further demonstrated the effectiveness of our proposed BSD-Net.

**Figure 5:**
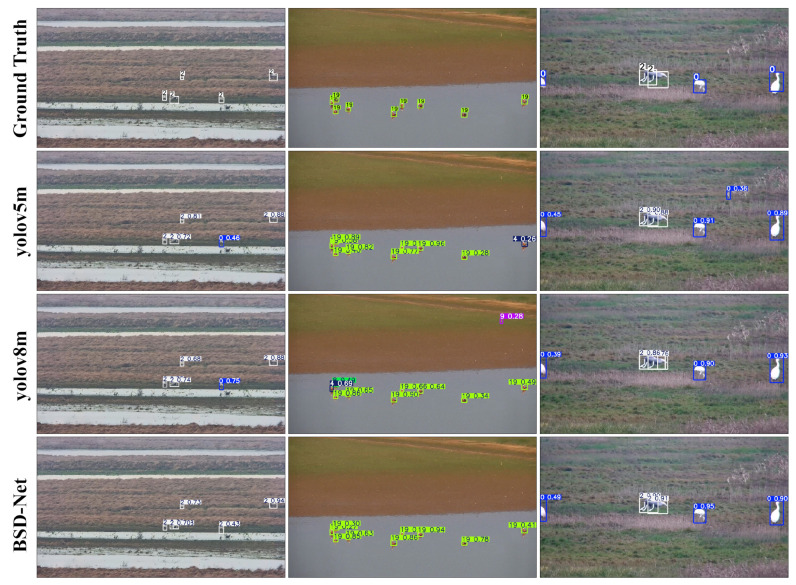
Comparison of the detection ability of different methods.

Evaluation on the Poyang Lake Bird dataset: Unlike the CUB200-2011 and FBD-SV-2024 bird datasets, the Poyang Lake Bird dataset contains multiple birds per image. Table 3 presents the quantitative performance of BSD-Net and other advanced networks on the Poyang Lake Bird dataset. The experimental results showed that BSD-Net outperformed other networks. Notably, BSD-Net’s mAP50 was 1.13% higher than yolov8m’s. In addition, the detection effects of YOLOv5, YOLOv8, and the proposed BSD-Net on the Poyang Lake Bird dataset are shown in Figure 5. The detection accuracy of the BSD-Net was higher than that of the other methods, and the false detection rate of non-avian objects was lower than that of the other methods.

### 4.4. Ablation Experiments

To further validate the effectiveness of the BSD-Net proposed in this paper, two ablation experiments were conducted to examine the impact of each module in the BSD-Net on network performance, with an experimental setup consistent with Section 4.2.

**Figure 6:**
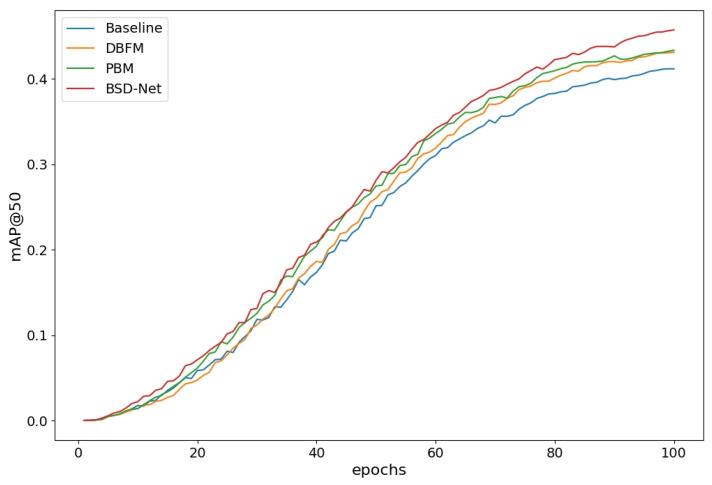
The experimental curve of the mAP50 of the core module.

Module ablation: We examined how the dual-branch feature mixer and prediction balance module affected the network performance using the CUB-200-2011 dataset. Figure 6 shows the training process for each module. Table 4 provides the quantitative results of the ablation experiments of various modules and shows that the network’s mAP50 increased by 2.2% with the addition of the prediction balance module to the baseline. It is worth noting that the number of parameters and FLOPs in the network did not increase. Therefore, PBM can aid in the training of higher-performing object detection networks. The addition of a dual-branch feature mixer to the baseline increased the network’s mAP50 by 1.8%. These results demonstrate that the dual-branch feature mixer improved the network’s feature extraction ability and allowed for efficient discrimination of locally similar features. However, the number of network parameters increased by 4.7 million, while the FLOPs increased by 17 G, but our experimental results demonstrated that the trade-off was worthwhile in terms of accuracy improvement. Between the baseline model and BSD-Net, the network’s mAP50 increased by 4.51%. It is worth noting that this performance boost came at only a minor increase in computational cost.

**Figure 7:**
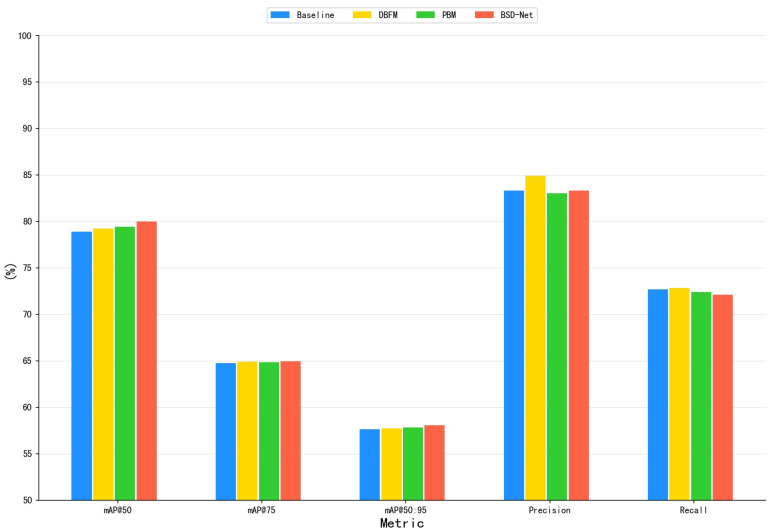
Results of different modules using the Poyang Lake Bird dataset.

To further evaluate the effectiveness of BSD-Net on multi-objective data, a modular ablation experiment was conducted on the Poyang Lake Bird dataset. Similar to the experimental results of the CUB-200-2011 dataset, the network performance was consistently improved, as shown in Table 5 and Figure 7. Adding the prediction balance module to the baseline increased the network’s mAP50 by 0.54%. Adding a dual-branch feature mixer to the baseline increased the network’s mAP50 by 0.35%. From the baseline model to BSD-Net, the network’s mAP50 improved by 1.13%.

Variance parameter ablation: Because the prediction equilibrium module has a unique hyperparameter variance, we investigated the effect of variance on the network performance. Table 6 provides the quantitative results for various variances using the CUB-200-2011 bird dataset. The experimental results showed that when the variance was 6, PBM had the least impact on the network performance, increasing the baseline mAP50 by 0.5%, while when the variance was 4, PBM had the greatest impact on the network performance, increasing the baseline mAP50 by 2.2%. It is worth noting that, while the variance was different, it had a minor impact on the network performance (the difference between the maximum and the minimum mAP50 was less than 2%), indicating that our PBM is somewhat robust with respect to hyperparameters.

### 4.5. Transfer Learning

To investigate the potential impact of transfer learning on the BSD-Net performance, the COCO object detection benchmark dataset was used for pretraining. The training process followed the 1× schedule (12 epochs) provided by the pyramid vision transformer [49]. Using the pretrained weights, the model was fine-tuned on the CUB-200-2011 dataset for additional 100 epochs. The quantitative results obtained using the CUB-200-2011 validation dataset are presented in Table 7 The results indicate that the use of pretrained weights could significantly accelerate the model convergence. It should be noted that even with pretrained weights, the BSD-Net outperformed the YOLOv8m model when using the CUB-200-2011 dataset.

### 4.6. CAM Visualization

**Figure 8:**
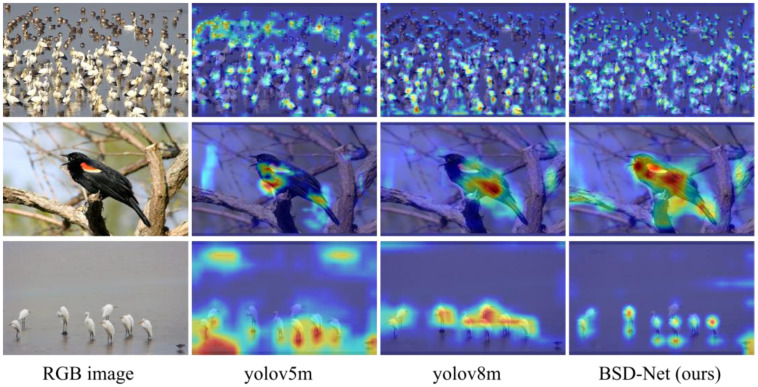
The CAM visualization results of the YOLOv5m, YOLOv8m, and the proposed BSD-Net using the CUB-200-2011 and Poyang Lake Bird datasets.

Next, to demonstrate the robustness of the obtained results, the class activation map (CAM) visualization results of the YOLOv5m, YOLOv8m, and the proposed BSD-Net using the CUB-200-2011 and Poyang Lake Bird datasets were analyzed, as shown in Figure 8. The results illustrated that the proposed BSD-Net could accurately identify the target locations of birds in the images and assign higher weights to the corresponding target regions. Furthermore, the proposed BSD-Net could effectively focus on targets of varying scales and quantities, thus enhancing detection accuracy.

## 5. Discussion

Our BSD-Net performed well on three challenging datasets. It is important to note that previous methods often fail to perform well on datasets with large (CUB-200-2011), small (FBD-SV-2024), and multiple targets (Poyang Lake Bird dataset) at the same time. However, with the help of a dual-branch feature mixer, BSD-Net not only achieved good detection results for both large and small targets, but also outperformed the previous method for multiple targets. We believe that there are a number of reasons for this: first, for large targets, DBFM can combine local and global information to identify similar local features, resulting in accurate bird species detection; second, for small targets, we believe that DBFM allows the network to extract more complete target structure features from the scene using fewer pixels; and finally, unlike the CUB-200-2011 and FBD-SV-2024 datasets, the Poyang Lake Bird dataset images often contain multiple birds of varying sizes, and there may be occlusion between pixels. BSD-Net combines global contextual information with the local details extracted by C2F, and feature fusion overcomes the limitations of a single local feature. For example, global context information can assist in distinguishing between large and small birds that overlap and are mistaken for a single bird.

In addition, through the ablation experiments of DBFM and PBM, we discovered that DBFM required approximately 4.5 million additional parameters during the training phase, whereas PBM required no additional parameters, implying that PBM can assist in training models with better performance and additional computational resources that are not required after deployment. It is important to note that PBM requires that the pixel values for each category be known. This condition is difficult to meet when performing unsupervised tasks. Therefore, it is worthwhile to investigate the design of a PBM appropriate for unsupervised tasks.

Overall, the advantages of the proposed BSD-Net in bird species detection in terms of accuracy make it a promising approach for avian biodiversity monitoring. For instance, Poyang Lake, which is an internationally important wetland, is home to the world’s largest bird sanctuary and a key habitat for migratory birds due to its wetland environment and favorable climate [50]. Every autumn and winter, over 98% of the global population of Siberian cranes migrate from Siberia to Poyang Lake to live and roost [51]. By deploying bird species detection models on monitoring equipment in the Poyang Lake area, this study realized round-the-clock, fully automated bird species detection and population counting. Compared to the manual monitoring methods, the proposed approach can significantly reduce time and effort. Particularly, bird species detection models provide frequent and consistent surveys. In addition, when these models are combined with monitoring equipment, they can promptly identify activities such as bird trapping and poaching, thus allowing for rapid conservation actions. Furthermore, the application of bird species detection methods in habitat protection is also important. Namely, by detecting the wintering areas of migratory birds each year, monitoring equipment can help generate species distribution maps, which are essential for identifying key habitats. Policymakers can use these maps to implement necessary conservation measures, such as harvesting sedges or releasing juvenile fish in key habitats before the arrival of migratory birds, thus ensuring an adequate food supply during the wintering period.

Future work could explore the integration of bird species detection data with multimodal data (e.g., environmental parameters and acoustic signals) to further enhance its practicality in bird conservation tasks.

## 6. Conclusions

In this study, we discovered that local similarity and category imbalance in birds are important factors in species detection. Therefore, we propose a new bird species detector called BSD-Net. The dual-branch feature mixer efficiently combines local details and global information to assist the network in distinguishing between locally similar bird characteristics. In addition, the proposed prediction balance module effectively balances the feature space differences caused by data imbalance. The experimental results using two public benchmark and a self-constructed Poyang Lake Bird datasets demonstrated that BSD-Net outperformed existing methods, achieving 45.71% and 80.00% mAP50 with the CUB-200-2011 and Poyang Lake Bird datasets, respectively, and 66.03% AP with FBD-SV-2024. In ablation experiments, our BSD-Net outperformed state-of-the-art object detectors, with clear advantages.

## Figures and Tables

**Table 1:** The quantitative results of different methods obtained using the CUB-200-2011 dataset.

Method	#Params. (M)	FLOPs (G)	mAP_50_	mAP_75_	mAP_50:95_	P	R
Yolov5m [44]	25.2	64.6	32.48	29.09	25.19	31.70	38.30
Yolov6m [45]	52.1	162.1	14.28	12.55	11.15	15.30	27.20
Yolov8m [35]	25.9	79.3	41.20	37.27	32.54	37.90	46.30
Yolov9m [46]	20.3	48.4	48.17	42.79	37.50	44.40	49.70
RT-DETR [24]	32.4	104.3	15.27	13.23	11.99	23.60	23.20
BSD-Net (Proposed)	30.6	96.3	45.71	44.54	34.64	41.90	48.20

**Table 2:** The quantitative results of different methods obtained using the FBD-SV-2024 dataset.

Method	Image Size	AP_50_	AP_75_	AP
SSD [47]	640 × 640	59.90	27.70	29.90
SELSA [48]	1000 × 600	40.00	16.20	19.30
Yolov5l [44]	640 × 640	55.80	30.70	29.90
Yolov6l [45]	640 × 640	58.50	30.80	30.40
Yolov8l [35]	640 × 640	58.40	32.70	31.80
Yolov9e [46]	640 × 640	57.70	32.20	31.80
FBOD-BMI [19]	672 × 384	69.20	30.20	35.10
RT-DETR [24]	640 × 640	62.14	25.21	29.15
BSD-Net (Proposed)	672 × 384	66.03	38.92	36.75

**Table 3:** The quantitative results of different methods obtained using the Poyang Lake Bird dataset.

Method	#Params. (M)	FLOPs (G)	mAP_50_	mAP_75_	mAP_50:95_	P	R
Yolov5m [44]	25.2	64.6	77.12	61.78	55.08	80.70	70.60
Yolov6m [45]	52.1	162.1	66.69	52.27	46.72	75.70	63.70
Yolov8m [35]	25.9	79.3	78.87	64.73	57.64	83.30	72.70
Yolov8-p2 [35]	25.6	98.7	78.44	65.34	58.05	86.70	72.30
RT-DETR [24]	32.8	108.1	51.95	37.54	33.71	75.90	49.70
BSD-Net (Proposed)	30.6	96.3	80.00	64.94	58.06	83.30	72.10

**Table 4:** Quantitative results of ablation experiments with different modules.

DBFM	PBM	#Params.(M)	FLOPs (G)	mAP_50_	mAP_75_	mAP_50:95_	P	R
		25.9	79.3	41.20	37.27	32.54	37.90	46.30
√		30.6	96.3	43.00	38.69	33.90	40.70	44.90
	√	25.9	79.3	43.40	39.07	34.10	40.50	46.40
√	√	30.6	96.3	45.71	44.54	34.64	41.90	48.20

DBFM stands for dual-branch feature mixer, and PBM stands for prediction balancing module.

**Table 5:** Quantitative results of the ablation experiments with different modules (Poyang Lake Bird dataset).

DBFM	PBM	#Params.(M)	FLOPs (G)	mAP_50_	mAP_75_	mAP_50:95_	P	R
		25.9	79.3	78.87	64.73	57.64	83.30	72.70
√		30.6	96.3	79.22	64.91	57.73	84.90	72.80
	√	25.9	79.3	79.41	64.82	57.81	83.00	72.40
√	√	30.6	96.3	80.00	64.94	58.06	83.30	72.10

**Table 6:** Quantitative results of the ablation experiments with different variance.

Method	mAP_50_	mAP_75_	mAP_50:95_	P	R
w/o PBM	41.20	37.27	32.54	37.90	46.30
σ = 3	42.43	38.78	33.64	40.20	46.00
σ = 4	43.40	39.07	34.10	40.50	46.40
σ = 5	41.95	38.06	33.16	39.40	45.90
σ = 6	41.75	37.81	32.97	38.20	46.90

**Table 7:** The quantitative results of transfer learning obtained using the CUB-200-2011 dataset.

Method	Pre-Training Weight	mAP_50_	mAP_75_	mAP_50:95_	P	R
Yolov8m		41.20	37.27	32.54	37.90	46.30
BSD-Net (Proposed)		45.71	44.54	34.64	41.90	48.20
Yolov8m	**√**	69.91	66.43	60.28	66.50	65.60
BSD-Net (Proposed)	**√**	73.81	70.31	64.11	70.60	67.50

## Data Availability

Data will be made available on request.
